# Supplementary material for: HostSeq: a Canadian whole genome sequencing and clinical data resource
Source: BMC Genom Data. 2023 May 2;24:26. doi: 10.1186/s12863-023-01128-3 (PMC10152008; doi:10.1186/s12863-023-01128-3)
Supplement: Supplementary file 1 — Additional file 1: Table S1. HostSeq Core Consent Elements. In order to deposit datasets in HostSeq COVID-19 controlled-access Databank, all the elements in this table must be obtained in the research consent. Table S2. HostSeq Case Report Form. Table S3. Software used for processing WGS data. Table S4. List of HostSeq participating studies as described in respective protocols. Table S5. Distribution of sex and age across HostSeq studies (n = 9,427). SD: Standard deviation; IQR: interquartile range. Figure S1. Quality of HostSeq genomes. (A) Missing rate < 5%, (B) Contamination rate < 3%, (C) Mean coverage >10. Figure S2. Predicted population admixture and ancestry classification in HostSeq genomes. Each bar represents a genome. Proportion of African, East Asian and European ancestries is determined, and genomes classified into 8 ancestry groups using GRAF-pop. They are further categorized into 5 superpopulations: AFR - African and African-American, AMR - Latin American Asian and Latin American African, EAS - Asian-Pacific Islander and East Asian, SAS - South Asian, and EUR - European. 3% of genomes remain uncategorized. Figure S3. Genetic distances score of HostSeq genomes. The four genetic distances (GD1-4) scores from GRAF-pop represent the distance of each genome from several reference populations and are used to predict ancestry. Barycentric coordinates of GD1 and GD2 are used to predict admixture proportion of African, East Asian and European ancestries. [file 12863_2023_1128_MOESM1_ESM.docx]

**APPENDIX**

Table S1. HostSeq Core Consent Elements. In order to deposit datasets in HostSeq COVID-19 controlled-access Databank, all the elements in this table must be obtained in the research consen**t.**

| **ELEMENTS** | **DETAILS** |
| --- | --- |
| **Research data** | Whole genome sequencing of the sample and the ongoing collection of clinical data from participant’s medical records/chart, administrative databases, etc. |
| **International sharing** | International sharing of genetic and clinical data |
| **Future research use** | Future health research on COVID-19 and other health outcomes |
| **Commercial use** | Use of genetic and clinical data for commercial purposes |
| **Controlled access** | Sharing of genetic and clinical data through a controlled-access mechanism |
| **Storage on cloud servers in Canada** | Storage of genetic and clinical data in the HostSeq Databank, on centralized Canadian cloud servers |
| **Duration of storage** | Indefinite storage of genetic and clinical data |
| **Data withdrawal** | Not possible to withdraw data that has already been distributed and used |
| **Re-identification** | Low risk that the participant could be re-identified in the future |
| **Re-contact (optional)** | Optional re-contact to update personal information, provide new health information, or to be invited to participate in new research projects. |

Table S2. HostSeq Case Report Form.

| **IDENTIFICATION** | **COMORBIDITIES** | **LAB RESULTS** |
| --- | --- | --- |
| Host Hospital | **Comorbidities: Immune system** | Haemoglobin |
| Source study participant ID | HIV | WBC count |
| HostSeq ID | Immunocompromised status | Lymphocyte count |
| DNA ID | Onset of COVID-19 to immunocompromisation | Neutrophil count |
| Sequencing site | Organ transplant | Haematocrit |
| Was informed consent obtained? | Organ type of transplant | Platelets |
| Study enrollment date | Autoimmune or rheumatologic disease | APTT/APTR |
| DOB | Diabetes | APTT |
| Age | Type I diabetes | PT |
| Laboratory COVID-19 test result | Type II diabetes | INR |
| Suspected to be COVID-19 positive? | **Comorbidities: Respiratory system** | ALT/SGPT |
| Ambulatory | Asthma | Total Bilirubin |
| Hospitalized State | COPD | AST/SGOT |
| Date of admission | Cystic fibrosis | Glucose |
| Sex at birth | Sleep apnea | Blood Urea Nitrogen (urea) |
| Gender | Do you use a home CPAP device? | Lactate |
| Ancestry | **Comorbidities: Genitourinary/Metabolic** | Creatinine |
| Height (cm) | Chronic kidney disease | Sodium |
| Weight (Kg) | Liver disease | Potassium |
| **DEMOGRAPHICS** | Gallbladder | Procalcitonin |
| Country of birth | Pancreas | CRP |
| Education | **Comorbidities: Cardiovascular system** | CT chest |
| Employment | Coronary intervention | Chest X-Ray performed? |
| Type of residence | Coronary artery bypass | ECG |
| Household composition | Congestive heart failure | POCUS |
| **PREGNANCY** | Hypertension | Echocardiogram |
| Pregnancy - currently pregnant? | Myocardial infarction | LDH |
| **RISK FACTORS** | Myocardial infarction Type I | D-Dimer |
| Cigarettes | Myocardial infarction Type II | Fibrinogen |
| How many cigarettes daily? | Peripheral vascular disease | Ferritin |
| Vaping | Stroke | Triglycerides |
| Cannabis | Arrhythmias | IL-6 |
| **AT ADMISSION/ASSESSMENT** | **Comorbidities: Neurological** | CD4 |
| COVID-19 test date | Dementia | CD8 |
| Date of diagnosis | Neurological or neuropsychiatric disease | CD4/CD8 ratio |
| Serology | **Comorbidities: Cancer** | NT-proBNP |
| Serology positive test date | Currently diagnosed with cancer? | BNP |
| Date of last negative test | Patient age at diagnosis | Troponin |
| PCR | Leukemia | **TREATMENT** |
| Commercial serology test kit name | Lymphoma | ICU or High Dependency Unit admission? |
| Onset date of first/earliest symptom | Sarcoma | If yes, date of ICU admission |
| ER triage date at this facility | Carcinoma | If yes, date of ICU discharge |
| Transfer from another facility? | Myeloma | Prone ventilation |
| ABO blood type | Mixed types | Inhaled nitric oxide |
| Rh factor | Cancer location | Tracheostomy inserted |
| Home medications | Cancer treatment in the past 12 months | Extracorporeal support |
| BCG vaccine | Other comorbidities | RRT or dialysis |
| COVID-19 Vaccine? | **PATHOGEN TESTING** | Inotropes/vasopressors |
| Name of first dose vaccine | Pathogen testing done during this illness | OTHER intervention or procedure |
| Name of second dose vaccine | Influenza | **MEDICATION** |
| **SYMPTOMS** | Coronavirus | Antiviral agent |
| Cough | RSV | Azithromycin “Zithromax” |
| Days with cough | Adenovirus | Any other antibiotic |
| Difficulty breathing | Enterovirus | Corticosteroid |
| Fever | Bacteria | Antifungal agent |
| Days with fever | Other infectious respiratory diagnosis | Colchicine |
| Heart rate | Was there a physician diagnosis of pneumonia? | Chloroquine “Aralen” |
| Highest respiratory rate | Suspected non-infective | Hydroxycholorquine |
| Systolic blood pressure | **COMPLICATIONS** | Tocilizumab “Actemra” |
| Diastolic blood pressure | Viral pneumonitis | Kineret “Anakinra” |
| Oxygen saturation | Bacterial pneumonia | IVIG |
| Fatigue | Acute Respiratory Distress Syndrome | Plasma |
| Myalgia (general aches and pain) | Pneumothorax | Other COVID therapy |
| Runny nose | Pleural effusion | **OUTCOME** |
| Sore throat | Cryptogenic organizing pneumonia (COP) | Outcome |
| Loss of taste/smell sense | Bronchiolitis | Outcome date |
| Nosebleed | Meningitis / Encephalitis | Self-care ability versus before illness |
| Ear pain | Seizure | Repeat hospital visit within 30 days? |
| Wheezing | Stroke / Cerebrovascular accident | Date and reason |
| Chest pain | Congestive heart failure |  |
| Joint pain | Cardiac inflammation (mark all that apply) |  |
| Headache | Cardiac arrhythmia |  |
| Seizures | Cardiac ischaemia |  |
| Altered consciousness/confusion | Cardiac arrest |  |
| Abdominal pain | Coagulation disorder / Disseminated Intravascular Coagulation |  |
| Diarrhea | Anemia |  |
| Nausea/vomiting | Rhabdomyolysis / Myositis |  |
| Conjunctivitis | Acute renal injury/ Acute renal failure |  |
| Skin rash | Gastrointestinal haemorrhage |  |
| Other symptoms | Pancreatitis |  |
| Asymptomatic | Liver dysfunction |  |
|  | Hyperglycemia |  |
|  | Hypoglycemia |  |
|  | Inflammatory syndrome/Kawasaki Disease like |  |
|  | Other, please specify |  |

Table S3. Software used for processing WGS data.

| **PROCESS** | **SOFTWARE** | **VERSION** | **URL** |
| --- | --- | --- | --- |
| Sequence alignment | DRAGMAP | 1.3.0 | <https://github.com/Illumina/DRAGMAP> |
| Sorting alignments | Picard tools | 2.25.0 | <https://broadinstitute.github.io/picard/> |
| Genotyping | HaplotypeCaller | GATK 4.2.5.0 | <https://gatk.broadinstitute.org/hc/en-us/articles/4418062719899-HaplotypeCaller> |
| Joint-calling | GenotypeGVCFs | GATK 4.2.5.0 | <https://gatk.broadinstitute.org/hc/en-us/articles/4418054384027-GenotypeGVCFs> |
| HLA Class I typing | OptiType | 1.3.1 | <https://github.com/FRED-2/OptiType> |
| Sample contamination | VerifyBamID2 | 2.0.1 | <https://github.com/Griffan/VerifyBamID> |
| Ancestry and sex prediction | GRAF | 2.4 | <https://github.com/ncbi/graf> |
| Miscellaneous | GATK | 4.2.5.0 | <https://gatk.broadinstitute.org/hc/en-us/articles/4418051394587--Tool-Documentation-Index> |
|  | Samtools | 1.14 | <http://samtools.github.io/> |
|  | Bcftools | 1.11 | [http://samtools.github.io/ bcftools/bcftools.html](http://samtools.github.io/) |
|  | PLINK | 1.90 and 2.0.0 | <https://www.cog-genomics.org/plink/> |
|  | R | 3.6.3 | <https://cran.r-project.org/> |

Table S4. List of HostSeq participating studies as described in respective protocols.

| **STUDY, PI** | **OBJECTIVES** | **ELIGIBILITY** |
| --- | --- | --- |
| GENCOV  Jordan Lerner-Ellis Jennifer Taher | To identify the characteristics of the antibody response that result in maintained immune response and better patient outcomes; to determine impact of genetic differences on COVID-19 infection severity and immune response; to determine impact of different viral strains on antibody response and patient outcomes. | All outpatients (seen in ER and assessment centres) with mild symptoms as well as hospitalized patients with severe symptoms, recruited from six hospitals in Ontario. |
| GenOMICC  David Maslove | To identify genetic determinants of severe, life-threatening COVID-19 infections. | All patients with confirmed COVID-19 admitted to ICU |
| CANCOV  Angela Cheung  Margaret Herridge | To evaluate early to one-year outcomes in patients with COVID-19 and their family caregivers. | Hospitalized patients (>16 years and positive COVID-19 test) and their caregivers.  Anticipated deaths or withdrawal of life-sustaining treatment within 48 hours, catastrophic neurological injury, patients unlikely to comply with follow-up are excluded. |
| genMARK  Upton Allen | **Primary**. To identify genetic variation associated with:  i). Susceptibility to COVID-19 and SARS-CoV infection among study subjects vs controls  ii). Likelihood of severe disease among study subjects.  **Secondary.** To assess immune responses,  including T cell function and cytokine/chemokine profiles among subjects with different severity of COVID-19. | Individuals without a known immunodeficiency who experienced illness due to confirmed COVID-19 infection.  Controls: individuals exposed to COVID-19 but are symptom-free as far as COVID-19 is concerned. Controls are tested for subclinical infection. Controls are 1:1 matched by age groups from within the same home, using the most distantly related household member. |
| LEFT-GEN  Juthaporn Cowan | To understand immunopathogenesis of persistent symptoms | All patients with confirmed COVID-19. |
| BQC19  Vincent Mooser | To support the institutions of the health and social services network (HSSN) by making the unique biological material and data accessible for research on COVID-19 and related diseases. | All patients who tested for COVID-19 at participating institutions |
| CONCOR-Donor  Rulan Parekh | To determine if the titers of SARS-CoV-2 specific neutralizing antibody and serum ELISA antibodies are correlated with clinical and demographic factors; to characterize duration of immunity over 1 year and determine if the titers are modified by clinical and demographic factors; to identify genetic predictors of SARS-CoV-2-specific neutralizing antibody and serum ELISA antibody titers at baseline and over time | Individuals ≥17 years, who recovered from COVID-19 infection and volunteer to donate convalescent plasma |
| AB3C  Francois Bernier | To establish prospective cohort study of all children in Alberta tested for or diagnosed with confirmed or probable COVID-19 infection; to conduct a detailed multiomic precision-medicine evaluation of some children in Alberta with confirmed or probable COVID-19 infection and some healthy controls; to evaluate adaptive immune response to SARS-CoV-2 virus in children measuring the development of immunity against COVID-19 in children with and without clinically apparent confirmed or probable COVID-19 infection | All children in Alberta, aged 0-17 years, who have a respiratory sample tested for COVID-19 in 2020; diagnosed with probable COVID-19 (as defined by Alberta Health), but not tested.  Controls: 25 healthy children in Calgary who are matched on age and gender. The controls will be identified from previous research conducted by the ACH Infectious Diseases Epidemiology & Vaccine Evaluation or enrolled in All Our Families cohort or in the Healthy Infants and Children Clinical Research Program. |
| Host Genetic Factors Underlying Severe COVID-19  Catherine Biggs | To determine what proportion of patients with severe COVID-19 have an underlying IEI and characterize the role of genetic factors in COVID-19 disease; to explore molecularly-targeted treatments e.g., if patients with hyperinflammation have variants dysregulating JAK-STAT signaling, JAK inhibitors | Individuals aged <70 years with documented COVID-19 infection, who are previously healthy with severe COVID-19. (Severe disease is defined as any of the following: requiring ICU admission, cardiorespiratory support such as non-invasive or mechanical ventilation, extracorporeal membrane oxygenation, or unusual complications such as multisystem inflammatory syndrome in children (MIS-C) associated with COVID-19, encephalitis, other inflammatory). |
| GD-COVID  Stuart Turvey | To identify host and viral genomic factors associated with COVID-19 disease susceptibility and health outcomes | Individuals of any age with documented SARS-CoV-2 infection, who are a resident of BC; have viral genome sequencing data available through VirusSeq; provide permission to contact Health Authority, CTCR or BCCDC COVID-19 survey |
| Understanding Immunity to Coronaviruses  Mario Ostrowski | To determine B cell and antibody immunity to coronaviruses; to identify candidate targets of the virus for vaccine development; to identify novel neutralizing antibodies to 2019-nCoV that can be used in therapeutics; to understand the innate immune response against 2019-nCoV; to identify host factors that play critical roles in developing strong immunity to coronaviruses. | Individuals 18 years old or older. Those with blood samples hemoglobin < 100 g/L or pregnant or with hemophilia or platelet count < 50,000 / ul are excluded. |
| SickKids COVID-19 Biobank  Rae Yeung | To establish a central biobank of biological samples and related core patient level data that will serve as a single point of access that supports a unified and collaborative approach to clinical and translational research related to the current COVID-19 pandemic and future works. | All patients presenting to the Hospital for Sick Children with a suspected or confirmed diagnosis of COVID-19, and family members of participants as applicable. |
| AB-HGS    Gerald Pfeffer | Identify host genetic susceptibility factors associated with requirement for hospitalisation due to COVID-19.  Family members who did not require hospitalisation were recruited as controls. | Confirmed SARS-CoV-2 infection (by provincial nucleic acid test)  Required admission to hospital (either medical units or critical care)  Age <60 years  Unvaccinated participants only  Omicron variant cases were excluded |

Table S5. Distribution of sex and age across HostSeq studies (n=9,427). SD: Standard deviation; IQR: interquartile range.

| **STUDY** | **SEX** | | **AGE** | |
| --- | --- | --- | --- | --- |
|  | Male | Female | Mean (SD) | Median (IQR) |
| GENCOV | 515 (46.4%) | 596 (53.6%) | 45.3 (14.6) | 44.0 (23.0) |
| GenOMICC | 179 (54.1%) | 152 (45.9%) | 63.5 (14.4) | 66.0 (18.5) |
| CANCOV | 558 (42.8%) | 746 (57.2%) | 51.8 (16.1) | 51.0 (24.0) |
| CONCOR-Donor | 241 (30.2%) | 557 (69.8%) | 44.5 (13.5) | 44.0 (22.0) |
| BQC19 | 1,744 (46.4%) | 2,011 (53.6%) | 52.5 (20.6) | 52.5 (29.5) |
| genMARK | 170 (28.5%) | 426 (71.5%) | 36.9 (15.8) | 38.0 (21.0) |
| LEFT-GEN | 23 (52.3%) | 21 (47.7%) | 50.9 (15.5) | 50.0 (24.3) |
| GD-COVID-19 | 308 (38.8%) | 485 (61.2%) | 46.3 (18.9) | 46.0 (28.0) |
| Host Genetic Susceptibility | 36 (50.7%) | 35 (49.3%) | 49.5 (11.4) | 52.0 (16.0) |
| Understanding immunity | 5 (50.0%) | 5 (50.0%) | 43.6 (13.9) | 42.5 (21.0) |
| AB3C | 95 (50.5%) | 93 (49.5%) | 11.5 (4.5) | 12.0 (7.0) |
| SCB | 144 (64.6%) | 79 (35.4%) | 9.6 (7.0) | 8.0 (13.0) |


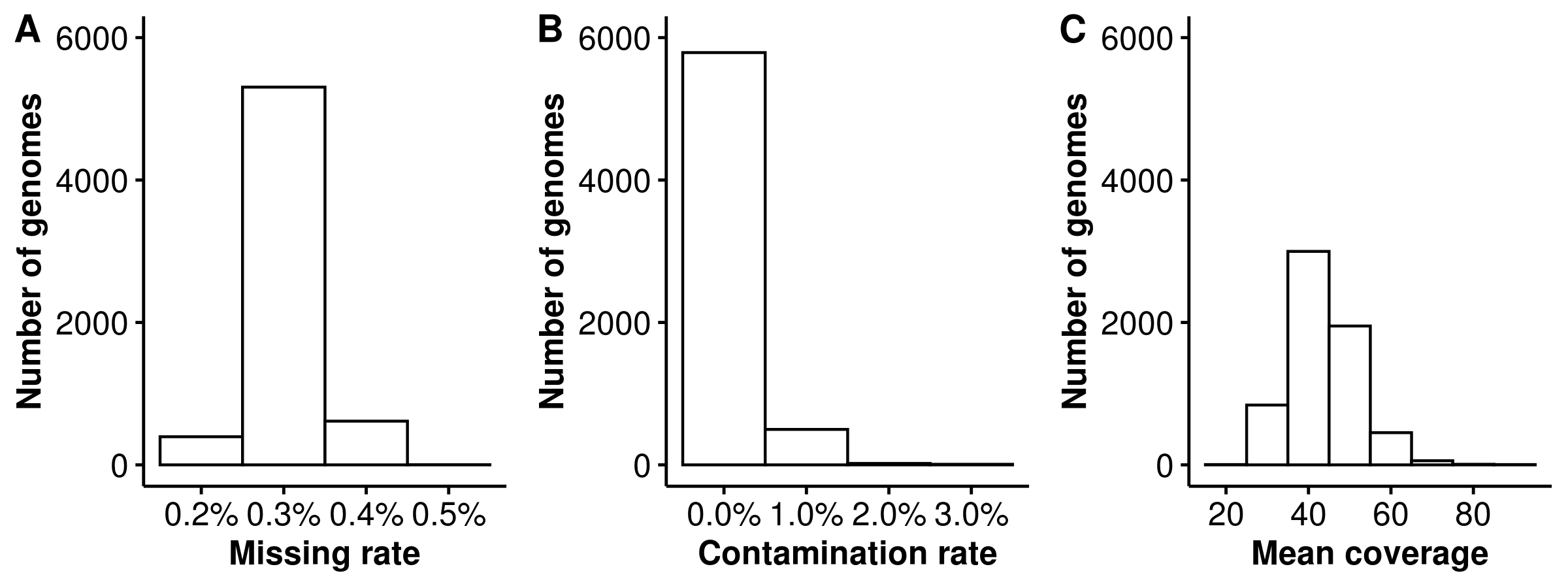


Figure S1. Quality of HostSeq genomes. (A) Missing rate < 5% (B) Contamination rate < 3% (C) Mean coverage >10.


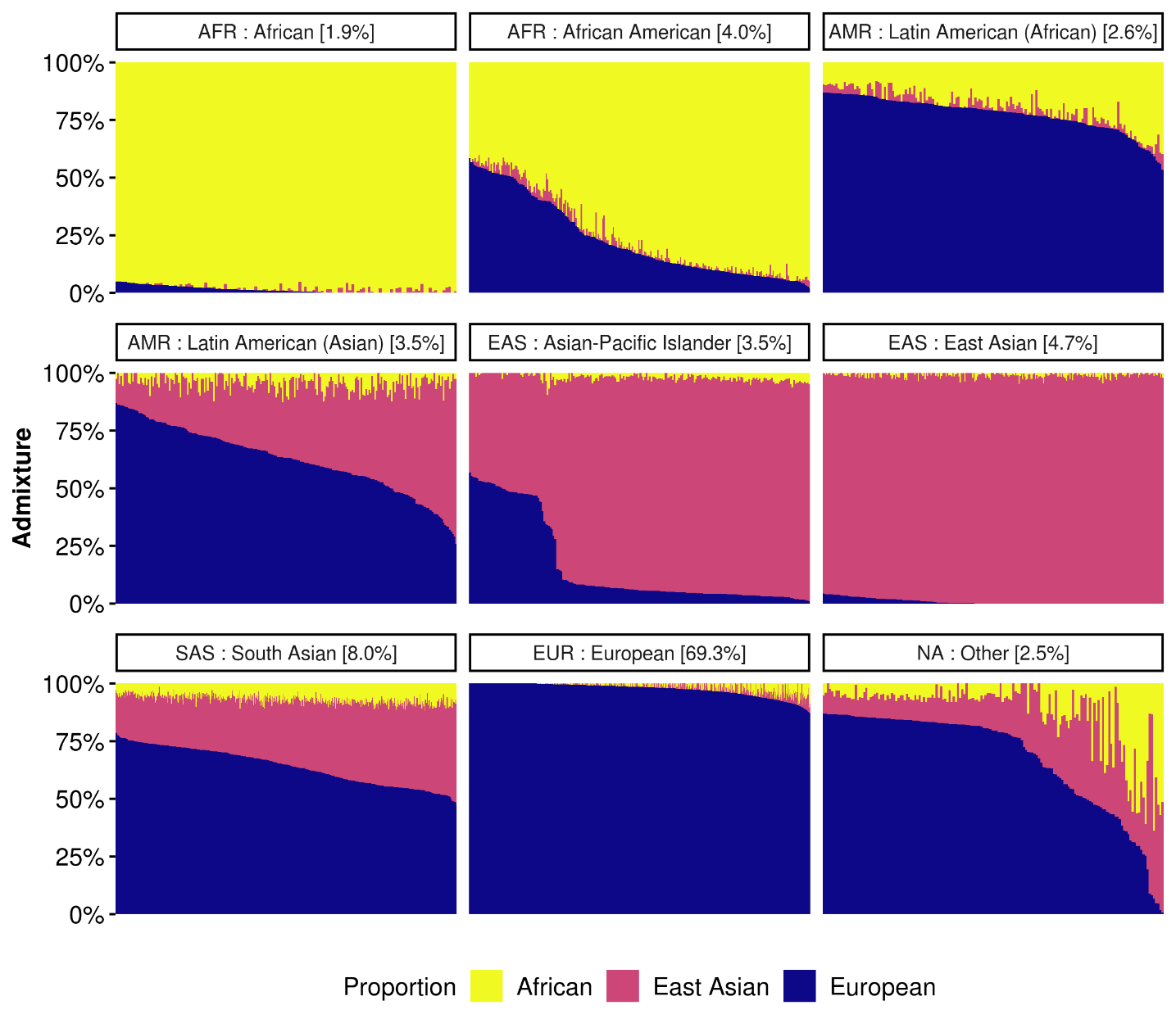


Figure S2. Predicted population admixture and ancestry classification in HostSeq genomes. Each bar represents a genome. Proportion of African, East Asian and European ancestries is determined (according to their definitions in GRAF), and genomes classified into 8 ancestry groups using GRAF-pop (see Methods). They are further categorized into 5 superpopulations: (i) AFR - African and African-American, (ii) AMR - Latin American Asian and Latin American African, (iii) EAS - Asian-Pacific Islander and East Asian, (iv) SAS - South Asian, and (v) EUR - European. 3% of genomes remain uncategorized.


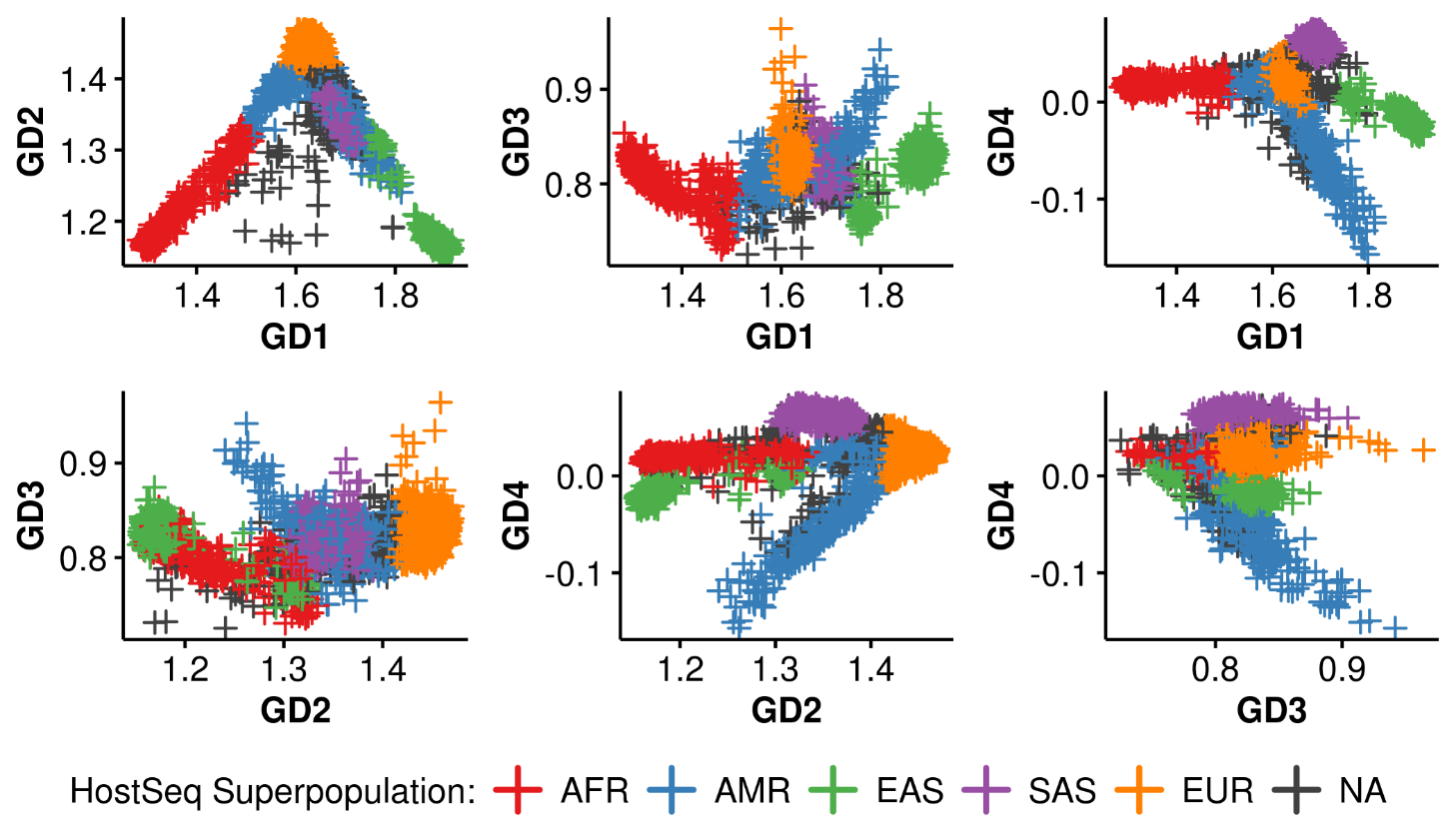


Figure S3. Genetic distances score of HostSeq genomes. The four genetic distances (GD1-4) scores from GRAF-pop (see Methods) represent distance of each genome from several reference populations and are used to predict ancestry. Barycentric coordinates of GD1 and GD2 are used to predict admixture proportion of African, East Asian and European ancestries.
